# St. Louis Obstetrical and Gynecological Society

**Published:** 1881-02

**Authors:** 


### ST. LOUIS OBSTETRICAL AND GYNECOLOGICAL SOCIETY.

Copied from the Obstetrical Gazette.

*Vomiting in Pregnancy.*—Dr. Moses: Vomiting in pregnancy depends on either a peculiarly vicious condition of the system, or upon uterine irritation. It may depend on a deranged stomach, or as it very often does, upon excoriation of the neck of the uterus, and any of the simple plans of treatment will be successful. I have seen several cases in which a single application to the os gave relief. At times laudanum or chloral enemata may be used with good effect, and I have seen leeches over the stomach answer the purpose; but I have seen everything fail. I have had cases myself, and have seen others in consultation in which all these measures were used without avail, and not until natural or artificial abortion had been induced, did the patient find relief. The relief was instant on the death of the fetus. About four or five years ago, I was called in consultation with Dr. McFeeters to the case of a woman suffering with continuous vomiting for six weeks, until she was on the verge of starvation. Every means which I have spoken of was tried in this case and without the slightest effect; she vomited all the time, retaining nothing whatever. Nutrient enemata had no effect. Finally I concluded that it was justifiable to produce abortion. A sound was introduced between the membrane and uterine walls with the result of at once arresting the nausea; abortion was so tardy that I almost doubted whether she was pregnant. It was ten days after introduction of the sound before she cast off the embryo. While this is a dernier resort, I think it justifiable where all the means which are known have been tried in vain. I consider it criminal to allow a patient to starve herself to death if it is possible to relieve her, and I would not hesitate one instant when all other means had failed, to bring on an abortion. I have done it three times, once without avail, for the patient was dying at the time of the operation. In another case everything that was known was done to relieve this symptom without avail, and as the woman was starving to death, I produced abortion; she got perfectly well, afterwards giving birth to four or five living children without any great amount of nausea during pregnancy. With regard to the case which Dr. Robinson relates with so many interesting details, I myself recall a case somewhat similar, which did not occur in the case of a person reduced from nausea. She was a healthy, vigorous woman, perhaps a little inclined to be fat. She never had any organic disease; she was in fine health; her labor was an ordinary one. In a couple of days a condition came on and continued for six or eight weeks, such that this lady could not possibly be moved in any way; we could not lift her feet without her complaining. If her head was raised from the pillow, she fell into a condition of coma. In order to change the sheets, which was required regularly, we slid them under her. She was stimulated, and fed with three pounds of beef broth every twenty-four hours. Whether this case was dependent on heart clots or not I am unable to say, but think now it was. The patient is living now in fine health. In this case there was no peripheral thrombosis.

Dr. Prewitt: I think Dr. Robinson's view of it, so far as establishing the heart clot is concerned, is probably a correct one. The condition was certainly such as favored the formation of heart clot. The symptoms were such as to point to this condition. You will recall that we are indebted to Dr. B. W. Richardson, of London, for the investigation of this subject, a very admirable treatise on the symptoms attending heart disease, in which are formulated the symptoms. The patient was in such condition as would result if bled profusely. Dr. Richardson goes to the extent of showing that we can not only make a diagnosis, but that it can be determined upon which side of the heart the clot is located, that the heart sounds are influenced by the pressure of the clot, and, if the clot is upon the left side, for instance, the sound will be greatly diminished or absent, while on the right side they could be heard. Now if the heart clot could be dissolved gradually, it could be done probably without ill consequences; but if it be detached in fragments, there would be emboli; and Richardson cites a case where he had gone out of London to see a patient in consultation. The patient was almost moribund. He made a diagnosis of heart-clot on the left side. There was one chance for the patient to live, and that was the loosening of the clot and it floating away. Of course, if it did so, there would be embolism somewhere. During the night this thing occurred, the patient was relieved, and there was evidence of the embolism of the fermoral artery. The patient recovered. Now in these cases where the heart-clot is large, and relief dependent on its dissolution, I should imagine the chance would be very small; in case it had been detached and had blocked the pulmonary artery, of course death would have been instantaneous, whereas having occurred upon the left side in the case which Dr. Richardson cites, having taken the direction of the leg instead of the brain, the patient recovered with the symptoms of embolism of the artery of the leg.

Dr. Boisliniere: I wish Dr. Robinson would report the case he saw in connection with me, the case of the lady who had puerperal phlebitis. At one time she began to have a cough, and then he was called in; there was a sudden occurrence of eclampsia. Eclampsia begins, usually, with a spasm of the small muscles of the face, and finally the large muscles. This case did not begin thus. It began by violent convulsions. She occasionally complained of intense pain in the back of the head. A diagnosis was made of thrombosis occurring in the right side of the heart, followed by pulmonary embolism and later, embolism of the cerebral arteries.

Dr. Robinson: There is not much more to say in regard to the case, but it resembled very much a case I saw occurring in a young man about twenty years of age who had thrombosis of the left fermoral vein with inflammation of the vein; he suffered from abdominal trouble, apparently the result of embolism. That was followed by an embolism of the cerebral artery upon the left side and hemiphlegia on the right side of the face and body. The case was strikingly like that of the lady whom Dr. Boisliniere referred to. There is one point in the case I reported, that should be noted, that is, although she had attacks of syncope, she did not lose consciousness entirely at all times. At times she would be in a state of most profound collapse, and then at other times she could speak only in whispers. She was so weak that she could not raise her arm or scarcely lift the fingers. During the severest attacks, I may say she had no muscular power at all. She was like the patient spoken of by Dr. Moses, being kept for weeks without anything under her head at all, and we changed the bed clothes by sliding them under her. I recall a case now, which Dr. Prewitt will, no doubt, recollect, in which there could be no other cause assigned but thrombosis, which ended in the death of my patient at the City Hospital some years ago. It was a thrombosis of the left femoral vein, and it extended up to the left bifurcation of the ascending cava. There was a much enfeebled heart action. We did not recognize the exact condition, for the pericardium was partially filled with pus. If I remember correctly, there was a perfectly sacculated condition of the pericardium, the sac communicating with the proper cavity of the pericardium, and it was filled with pus, which ran from one to the other. The cardiac action was very much diminished. The enfeebled circulation in this case may have been the immediate cause of the thrombosis.

Dr. G. A. Moses: I think the diagnosis made in this case is satisfactory; though at the time I witnessed the case in consultation very frequently, I couldn't help feeling great uncertainty. I think Dr. Robinson shared my feeling. The patient was extremely nervous. There were no symptoms pointing to any uterine trouble, no inflammatory complication, so far as regards the recovery of the genital organs after labor; but after this condition had continued for some time, with scarcely any change, one day at twelve o'clock I was called in haste, by reason of the discovery of blood in the bed, and upon the bed-pan. I found this discharge was not from the bladder, but from the uterus. Upon examination this proved to be the first return of menstruation. The patient has had no serious trouble since that date. It is a very peculiar coincidence that this return of the menstrual flow was normal, it only lasted the ordinary time, and produced no extraordinary symptoms, convalesence immediately commencing. The gradual solution of the clot takes place by very slow degrees necessarily, and although there is no other diagnosis by which to explain the condition satisfactorily, the case was accompanied throughout by very many singular associated symptoms.

sented to you, to-night, by our faithful and reliable recording secretary, Dr. G. A. Moses.

Permit me here to add that our work has met with the appreciation of the medical public, from which we have received encouraging praises. A highly appreciative com pliment has been offered us by the talented editor of the *Obstetric Gazette*, stating that our society had done the best work of any society of the kind in this country.

Allow me to compliment our society upon the spirit of scientific research, the high-toned courtesy in debate, and the sincere cordiality which have not ceased to lend their charms to our meetings and made them so attractive. There is every reason to believe that the same spirit will continue to prevail.

In closing these hasty remarks, I beg to be permitted to express to you my sincere thanks for your kind co-operation in assisting me to perform the duties of my office, and also to offer you my sincere wishes for your continued prosperity.

---

### Selections.

---

### "ANÆSTHESIA IN LABOR."

By DUDLEY M. CULVER, M.D., WHITESVILLE, INDIANA.

This is a subject that is attracting the attention of the profession to no small extent at the present day, and it is a subject that demands statistics of actual cases to establish its efficacy or the opposite. For young men of to-day, just starting in the practice of medicine, have many traps easily ensnaring them, and who can they look to for counsel and authority but the old members of the profession?

Anæsthetics have been used just enough now in labor to make a vast number of women appeal to the tender compassion of the accoucheur to administer chloroform. And they will tell the young physician that Dr. —— did use it on Mrs. ——, and no bad results followed, and it will

